# Size and structure of hexanuclear plutonium oxo-hydroxo clusters in aqueous solution from synchrotron analysis

**DOI:** 10.1107/S1600577521012005

**Published:** 2022-01-01

**Authors:** Thomas Dumas, Matthieu Virot, Denis Menut, Christelle Tamain, Cyril Micheau, Sandrine Dourdain, Olivier Diat

**Affiliations:** a CEA, DES, ISEC, DMRC, Univ Montpellier, Marcoule, France; b ICSM, CEA, Univ Montpellier, CNRS, ENSCM, Bagnols sur Cèze, France; c Synchrotron SOLEIL, L’Orme des Merisiers Saint Aubin, BP 48, 91192 Gif-sur-Yvette Cedex, France

**Keywords:** plutonium, clusters, SAXS, EXAFS

## Abstract

The size and shape of a water-soluble hexanuclear plutonium cluster were probed by combining synchrotron small-angle X-ray scattering (SAXS) and extended X-ray absorption fine structure (EXAFS). A specific setup coupling both techniques and dedicated to radioactive samples on the MARS beamline endstation at Synchrotron SOLEIL is described. The plutonium hexanuclear cores are well stabilized by the 1,4,7,10-tetraazacyclododecane-1,4,7,10-tetraacetic acid ligands and this allows a good evaluation of the setup to probe the very small plutonium core. The results show that, in spite of the constrained conditions required to avoid any risk of sample dispersion, the flux and the sample environment are optimized to obtain a very good signal-to-noise ratio, allowing the detection of small plutonium aggregates in an aqueous phase. The structure of the well defined hexanuclear cluster has been confirmed by EXAFS measurements in solution and correlated with SAXS data processing and modelling. An iterative comparison of classical fit models (Guinier or sphere form factor) with the experimental results allowed a better interpretation of the SAXS signal that will be relevant for future work under environmentally relevant conditions.

## Abstract 2

A water-soluble plutonium cluster made of six plutonium atoms decorated by four 1,4,7,10-tetraazacyclododecane-1,4,7,10-tetraacetic acid ligands is characterized by small-angle X-ray scattering (SAXS) and extended X-ray absorption fine structure (EXAFS).

## 1. Introduction

Because plutonium is highly radiotoxic, it represents one of the major long-term risks in nuclear waste management. To predict and control the behaviour of plutonium under various environmental conditions, thermodynamic models have been developed to describe plutonium solubility and surface inter­actions (Altmaier *et al.*, 2013 ▸; Geckeis *et al.*, 2013 ▸; Kersting *et al.*, 1999 ▸). Nevertheless, new experimental inputs are still necessary in order to improve the reliability of these models. Among them, any additional fundamental knowledge of the complex plutonium chemistry at the molecular scale is highly desirable for the safety of long-term nuclear disposal.

In this scope, and within the large variety of plutonium chemical and redox states, hydrolyzed forms of tetravalent plutonium are crucial objects to consider. Due to its high charge density in aqueous solution, the Pu^4+^ ion has a high tendency for hydrolysis reactions and can further polymerize through oxolation and/or olation reactions. The intermediate clusters formed through these reactions can further condense to yield colloidal plutonium nanoparticles which might enhance its mobility (Dalodière *et al.*, 2017 ▸; Gerber *et al.*, 2020 ▸; Micheau *et al.*, 2020 ▸; Neck *et al.*, 2007 ▸; Walther *et al.*, 2007 ▸; Walther & Denecke, 2013 ▸). Until now, the addition of complexing molecules was considered to limit the growth of these colloids, leading to smaller polynuclear species and affecting plutonium solubility in complexing humic acid conditions (Marsac *et al.*, 2014 ▸). Even though analogous tetravalent cations can also form polynuclear clusters with different numbers of metallic cations *M* ^4+^ in the nuclear core (Hennig *et al.*, 2012 ▸, 2017 ▸; Knope *et al.*, 2011 ▸; Takao *et al.*, 2012 ▸), recent work has only characterized small polynuclear plutonium clusters.

Knope & Soderholm (2013 ▸) first revealed the formation of solid Li_6_[Pu_6_(OH)_4_O_4_(H_2_O)_6_(HGly)_12_]Cl_18_·10.5H_2_O, which consists of a mixed hydroxo/oxo plutonium(IV) hexanuclear cluster. More recently, Tamain *et al.* (2016 ▸, 2017 ▸) used visible spectroscopy and X-ray absorption spectroscopy (XAS) techniques to correlate solid-state and solution structures of another hexanuclear complex exhibiting the same [Pu_6_(OH)_4_O_4_]^12+^ core, stabilized with the polyamino carboxylic acid DOTA (1,4,7,10-tetraazacyclododecane-1,4,7,10-tetraacetic acid). Visible absorption and reflectance spectra and extended X-ray absorption fine structure (EXAFS) measurements confirmed the stability of the hexanuclear core from the solid state to the solution. However, both spectroscopic approaches only provide plutonium-centred information and indicate that the plutonium hexanuclear core is preserved in solution.

To describe long-range interactions in solution, small-angle X-ray scattering (SAXS) was applied in combination with EXAFS in aqueous solution. SAXS offers a complementary image of the plutonium species in solution. This technique has already been used for the characterization of other actinide polynuclear compounds in solution. For instance, SAXS was used on U oxo-clusters by Martin *et al.* (2018 ▸) to follow U^IV^ hydrolysis, condensation and growth up to the formation of U-38 clusters. It was also applied to uranyl peroxo nanospheres (Falaise & Nyman, 2016 ▸; Dembowski *et al.*, 2017 ▸; Zhang *et al.*, 2019 ▸) and even to small hexanuclear uranium core clusters by Falaise *et al.* (2017 ▸).

Recently, SAXS measurements became available for plutonium samples on the MARS beamline and were used in combination with XAS to study plutonium colloids (Micheau *et al.*, 2020 ▸). Although the molecular structure of the hexanuclear DOTA plutonium clusters is well identified by single-crystal X-ray diffraction (XRD) and the ability of the MARS beamline to perform SAXS measurements on plutonium colloids was previously demonstrated, the present work takes the opportunity to probe the more challenging hexanuclear core clusters of plutonium in aqueous solution. The idea of probing the small hexanuclear cluster core of plutonium was twofold: (i) to assign as a test the ability of this setup to detect small and dilute objects in aqueous solution and propose a more detailed description of the setup, and (ii) to provide information on the external shape of hexanuclear plutonium clusters in solution, that were previously only defined by the first two coordination shells of the plutonium atom.

## 2. Materials and methods

### 2.1. Synthesis of the [Pu_6_(OH)_4_O_4_(H_2_O)_8_(HDOTA)_4_] cluster

The plutonium DOTA hexanuclear cluster was synthesized as previously reported (Tamain *et al.*, 2016 ▸). Protonated DOTA (47 mg) was dissolved in water (400 µl) with heating and 1 *M* HNO_3_ (80 µl) was added after complete dissolution of the ligand. The plutonium solution was added to the ligand solution ([Pu]/[*L*] = 1/5). The mixture was left overnight to give crystals of plutonium(IV) hexamer.

**Figure 1:**
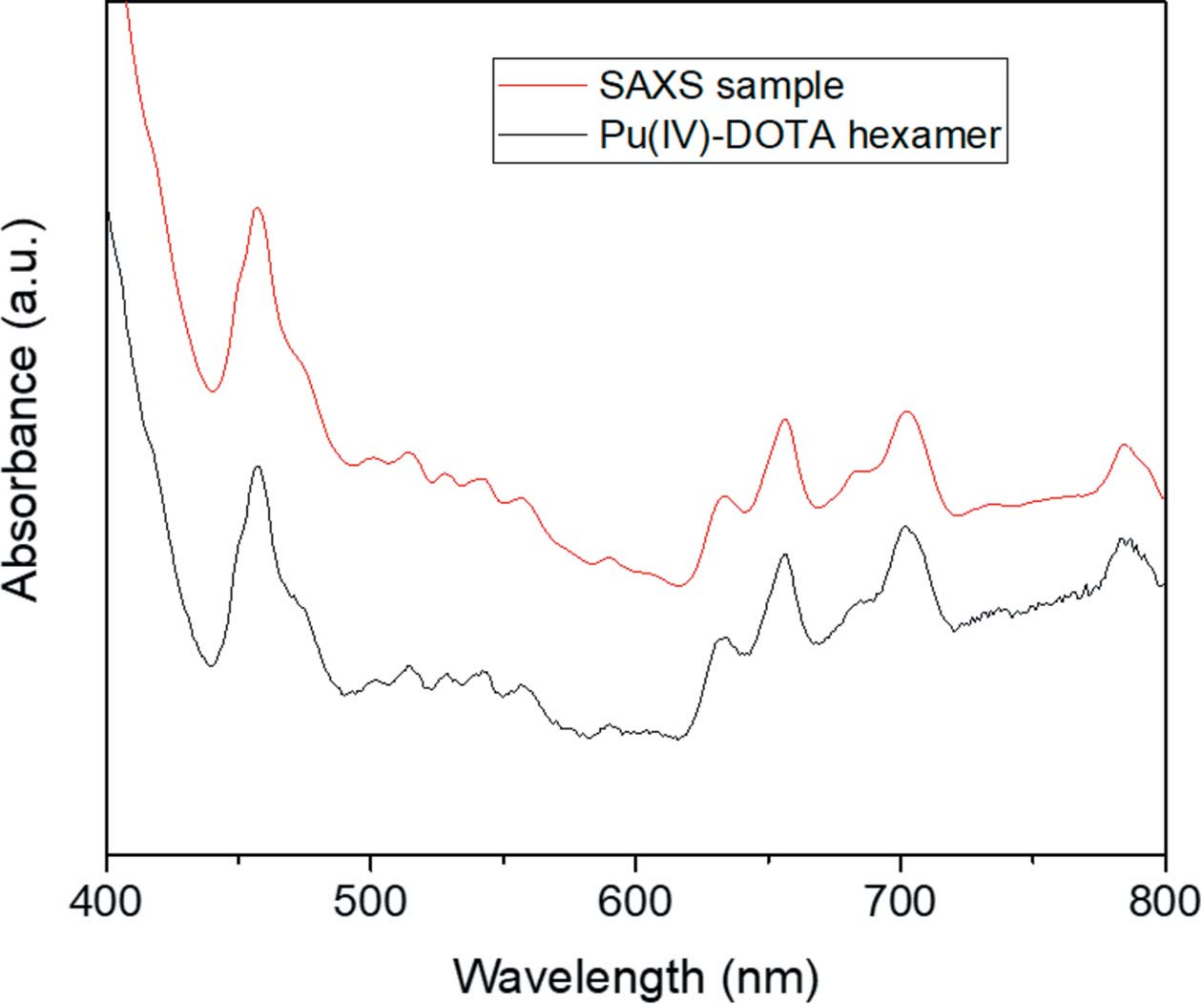
UV–Vis absorption spectrum of the SAXS–XAFS sample compared with the Pu^IV^–DOTA hexamer identified by single-crystal XRD.

The SAXS sample was prepared by dissolution of these crystals (2.3 mg) in 400 µL 1 *M* HNO_3_. The sample purity and concentration of 0.01 *M* (concentration of Pu involved in the hexamer) were checked using UV–Vis absorption spectroscopy in the visible range (400–800 nm) (Fig. 1 ▸). The spectrum collected in the visible range is identical to that reported previously and confirms the sample purity. The slight baseline deviation observed below 600 nm is related to a variation in HNO_3_ concentration between the two samples.

### 2.2. Measurement by synchrotron radiation and experimental setup

In this section, we give a brief summary of the design and relevant characteristics of the Multi Analyses on Radioactive Samples (MARS) beamline at Synchrotron SOLEIL (Saint-Aubin, France) (LLorens *et al.*, 2014 ▸; Sitaud *et al.*, 2012 ▸), as well as presenting more specific developments making the beamline especially suitable for scattering experiments.

From its inception, MARS was designed to accommodate the wide-ranging scientific requirements of the radionuclide and actinide sciences (up to highly radioactive materials) covering areas from biology and environmental science through to advanced materials for the direct characterization of their chemistry and microstructure with the use of synchrotron-based techniques. The beamline is licenced to receive samples with radioisotope activities up to two million times their isotope-specific European exemption limits and complies with all legal requirements and safety precautions to avoid contaminating aerosol dissemination and guarantee the public dose rate outside the beamline. It has successfully shown its flexibility by accommodating a wide array of diverse sample environments during its years of operation (Béchade *et al.*, 2012 ▸; Menut *et al.*, 2015 ▸; Béchade *et al.*, 2013 ▸; LLorens *et al.*, 2014 ▸; Dumas *et al.*, 2018 ▸; Bengio *et al.*, 2020 ▸).

X-rays are sourced from a 1.71 T bending magnet providing a continuous spectrum of photons with a critical energy of 8.6 keV. A double-crystal monochromator (DCM) (Oxford Danfysik, Oxford, UK) allows tunable monochromatization in the continuous energy range from 3.5 to 35 keV and horizontal focusing with a sagittal bender. The original feature of this DCM is its ability to exchange Si(111) and Si(220) crystal sets under vacuum to optimize the photon flux and energy resolution over the energy range. Two long mirrors are inserted before and after the DCM for high-energy harmonics rejection and to provide a vertically collimated and focused beam. These mirrors consist of silicon blocks with two different reflective surfaces: a 60 mm width silicon uncoated strip for energy setups below 14 keV and a 50 mm width platinum-coated strip (thickness 60 nm) for high energies. The X-ray beam at the second endstation of the MARS beamline (CX3), where the scattering and absorption measurements described here were performed, can be focused down to a spot size of 300 µm × 150 µm [full width at half-maximum (FWHM), horizontal (H) × vertical (V)], with the possibility of further microfocusing the beam down to 15 µm × 15 µm on the second endstation in the reduced range between 3.5 and 21 keV by inserting additional rhodium-coated mirrors in the Kirkpatrick–Baez geometry. Two kinds of two-dimensional detectors (image plate or hybrid pixel) are available for the scattering and/or diffraction analyses, which can be moved along the X-ray axis. Thus, depending on the energy used and the exact geometry of the scattering setup, the *Q* range achievable may be adapted.

As SAXS is contingent on the accurate subtraction of all background scattering contributions (optical elements, windows, slits, air *etc.*), reducing the effects of parasitic scattering is vital, not only to improve signal-to-noise ratios in the data but also to remove sources of scattering that may perturb the extraction of structural parameters. The instrument background on MARS was reduced by a standard three-paired-slits collimator system, where the first slits define the beam and adjust the flux, the second slits cut out the parasitic scattering fan coming from the optics and passing through the first pair of slits, and the third and last pair of slits remove the weaker secondary scattering produced by the second pair of slits. To reduce this background further, the third slits are set up with hybrid scattering slits from Xenocs (Sassenage, France). These scatterless slits are composed of heavy metal blades with Ge monocrystal lateral edges oriented far from any Bragg peak position with respect to the incident beam. Based on comparison of the energy-dependent attenuation lengths of Ge, it actually offers the best attenuation of energies above 12 keV for synchrotron-based SAXS instruments where they could yield even higher performance enhancements with the incident beam intensity (Li *et al.*, 2008 ▸). Extendable vacuum pipes sealed with Kapton windows are attached to the beamline to minimize the air gap around the sample position to a few centimetres and to keep the background scattering from air to a minimum, aided by motorized (*T_x_*, *T_z_*) sample stages with 1 µm resolution over a 100 mm range of motion. As radiation damage to samples, especially for solutions, may result in the formation of aggregates that can cause significant problems at a high-brilliance synchrotron source, the MARS beamline is equipped with a beam attenuator composed of eight pneumatic holders for aluminium foils of 0.05 to 8.0 mm thickness, allowing fine tuning of the beam attenuation to achieve the optimal balance between lowering the useful signal and reducing the effects of radiation damage.

**Figure 2:**
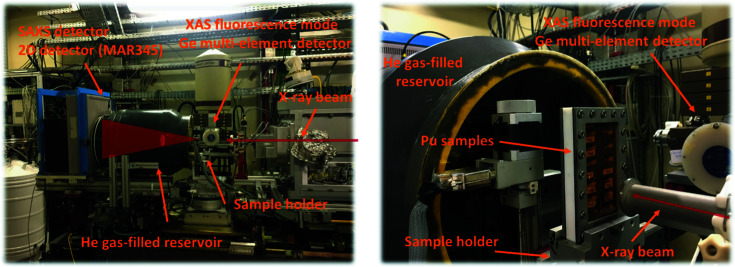
The MARS beamline CX3 endstation configuration to perform combined SAXS–XAS analyses. Reproduced with permission from Micheau *et al.* (2020 ▸) (ESI), copyright (2020) Royal Society of Chemistry.

Finally, a crucial step in SAXS data processing is the subtraction of the sample environment scattering from that of the sample, and these two independent patterns must be appropriately normalized to consider possible variation in the beam flux and differences in transmission. This operation can be conveniently accomplished by using an active beamstop. A miniature 3 mm × 2 mm active beamstop using a pin diode has been developed internally at SOLEIL. The beamstop has been shaped by additive manufacturing by tungsten laser powder bed fusion that made it possible to produce this complex functional component in a resource-efficient and economical manner. The beamstop is installed in the He-flushed flight tube which ends in a 345 mm-diameter Kapton window at the SAXS detector. The flight tube is positioned in the beam by motorized (*T_x_*, *T_z_*) stages. The ‘integration’ detector is a MAR-Research system with a sensitive phosphor imaging surface diameter of 345 mm (Fig. 2 ▸).

Combined SAXS and XAS analyses were performed with the storage ring operating in top-up mode at an electron current of 500 mA, 2.5 GeV. Beam energy calibration was performed at the yttrium *K*-edge, corresponding to 17.038 keV. The focused beam size on the sample was 300 µm × 400 µm (Fig. 2 ▸). A specific cell was used for radioactive samples and was composed of Teflon sample holders containing three slots of 250 µl closed by two layers of Kapton film (polyimide) on each side.

### 2.3. SAXS

SAXS experiments were carried out using the 2D image-plate detector (MAR345, marXperts GmbH, Germany), with an X-ray energy of 17 keV corresponding to an average wavelength of 0.7294 Å^−1^ and a sample-to-detector distance of 785.70 mm. The angular axis was calibrated using the powder diffraction pattern of silver behenate. This configuration allows coverage of a *Q* range between about 0.1 and 18.8 nm^−1^. Sample transmissions were recorded for 10 s using photodiodes and scattering diagrams were recorded for 5 s per sample. Isotropic 2D scattering data were azimuthally averaged to obtain the scattering intensity *I*(*Q*) using the *Fit2D* software (Hammersley *et al.*, 1996 ▸). The sample scattering intensity was then normalized by the acquisition time, transmission and thickness, and multiplied by a normalization constant determined with the help of the scattering intensity of polyethylene (PE) at *Q* = 0.36 nm^−1^ that corresponds to 4.9 cm^−1^. Finally, the absolute intensity scattered by the cluster was obtained by subtracting the empty cell, solvent and background contributions. Data were simulated using the *SasView 4.2.0* software (http://www.sasview.org) especially for core–shell, lamella and disc form factors.

### 2.4. Pu *L*_3_-edge XAS

Experimental spectra were recorded in fluorescence mode using a Ge multi-element detector (ORTEC, Oak Ridge, Tennessee, USA) at the Pu *L*_3_-edge. The samples were oriented at 45° with respect to the incident beam. The incident energy was calibrated using a metallic Zr foil (*K*-edge defined at 17.988 keV). The Pu ionization energy was defined at the maximum of the white line and corresponds to 18.068 keV. The spectrum corresponds to the average of six pre-processing scans obtained after 60 min of analysis each. EXAFS data were analysed with the *Athena* and *Arthemis* software from the *IFEFFIT* package (Ravel & Newville, 2005 ▸). Data fitting was performed using a Keiser–Bessel window with a cluster size of 1–5 Å for the Fourier transform (FT) in the 2 < *k* < 14 Å range [*Feff*, Version 8.4 (Ankudinov *et al.*, 1998 ▸)]. Two parameters were fitted, the atomic distances (*R*) and the Debye–Waller factor (DWF). Coordination numbers were fixed to the crystal structure value.

## 3. Results

**Figure 3:**
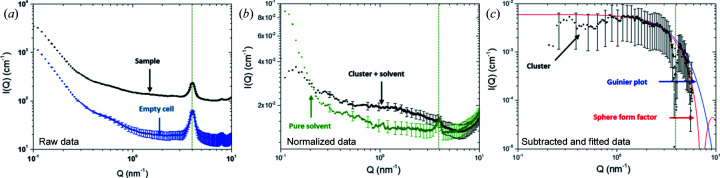
SAXS data for the plutonium cluster solution at different processing steps. (*a*) Raw data for the sample (black) and empty cell (blue) in arbitrary units (a.u.). (*b*) Normalized data in absolute units after empty-cell subtraction for the cluster sample (black) and the pure solvent (green). (*c*) The cluster signal (black) after subtraction of the solvent contribution, and the fitting models: sphere form factor (red line) and Guinier plot (blue line). The vertical dotted line in each graph corresponds to the expected position of the Kapton scattering peak.

The SAXS data processing operations performed on the signal acquired from the plutonium cluster are summarized in Fig. 3 ▸. The raw data curves in Fig. 3 ▸(*a*) correspond to the signal obtained from the sample and an empty cell as reference. Both diagrams exhibit a well defined peak at 4 nm^−1^ which is attributed to the scattering of the four Kapton layers on the sample cell. This peak is still visible after subtraction of the empty cell, as can be seen in Fig. 3 ▸(*b*) where the pure solvent and the sample are compared after normalization of the data according to the sample thickness, transmission, acquisition time and scattering intensity of polyethylene (PE) used as reference. The residual presence of the Kapton peak can be attributed to the non-reproducible handmade cell and to the impossibility of measuring the exact empty cell used for the radioactive sample. Nevertheless, both diagrams demonstrate a significant scattering difference when comparing the hexanuclear cluster and the solvent, particularly around 10^0^ nm^−1^. The absolute intensity scattered by the cluster is shown in Fig. 3 ▸(*c*) after subtracting the solvent contribution while considering the total conversion of Pu^IV^ into DOTA polyamino carboxylate PuO_2_ clusters (corresponding to a volume fraction of 0.018). Note that a subtraction artefact related to the Kapton is still visible at 4 nm^−1^. Small intensity variations are also observed at low *Q*. This can be attributed to a subtraction artefact due to the low scattering intensity of such dilute samples, or to a structure factor linked to cluster interaction or aggregation. Further investigations at higher concentrations are planned in order to investigate this diagram region. Nevertheless, the normalized SAXS diagram of the plutonium clusters [Fig. 3 ▸(*c*)] in absolute units can be fitted using simple models. This diagram displays a *Q* ^0^ slope at low wavevectors and tends to a *Q* ^−4^ slope at higher wavevectors, which behaviour is a typical signature of three-dimensional and compact objects with a sharp interface.

The scattering intensity is generally described as the product between an initial intensity *I*_0_ (when *Q* tends to 0), a form factor *P*(*Q*) relative to the shape of the solute, and a structure factor *S*(*Q*) related to the interaction between solutes (Glatter & Kratky, 1982 ▸). To a first approximation, and considering a very dilute solution, *S*(*Q*) is considered equal to 1. Two approaches were employed in the 1–6 nm^−1^ *Q* range to determine the size of the plutonium clusters: (i) Guinier analysis and (ii) a monodisperse sphere form factor fitting [Fig. 3 ▸(*c*)]. In these two models, the initial intensity *I*_0_ is defined as

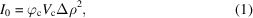

with 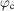 the volume fraction of the clusters, 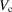 the volume of one cluster and 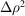 the scattering length density contrast with the solvent. Table 1 ▸ summarizes the scattering length density ρ_c_ and the contrast Δρ^2^ used for the calculations.

The Guinier analysis gives a first estimation of the cluster radius of gyration *R*_g_. A radius of gyration of around 0.48 nm is found. Considering spherical and filled entities, the radius of the cluster can be deduced with the following equation [equation (2)], where *R* is the radius of the sphere. This leads to a sphere radius of 0.62 nm (*cf.* 1.22 nm diameter).

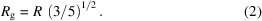

The best fit obtained with the monodisperse sphere form factor model is consistent with a sphere radius *R* of about 0.62 nm, which is in full agreement with the results obtained with the Guinier analysis. The fitting parameters are summarized in Table 2 ▸.

**Figure 4:**
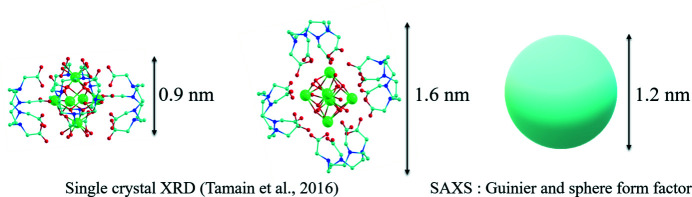
Comparison of the SAXS Guinier and sphere form factor models with the [Pu_6_(OH)_4_O_4_(H_2_O)_8_(HDOTA)_4_] complex structure from single-crystal XRD (Tamain *et al.*, 2016 ▸). The complex structure, in the solid state, shows the anisotropic DOTA complexation on the octahedral [Pu_6_(OH)_4_O_4_]^12+^ core which results in an oblate spheroid.

Finally, both models concur in the description of the cluster as nearly spherical particles measuring 1.2 nm in diameter in nitric acid solution. The size probed with the SAXS approach using a monodisperse sphere form factor may be compared with the one expected from the crystallographic data of the plutonium hexanuclear clusters (Tamain *et al.*, 2016 ▸). The [Pu_6_(OH)_4_O_4_(H_2_O)_8_(HDOTA)_4_] complex is made of a [Pu_6_(OH)_4_O_4_]^12+^ core decorated with four DOTA polyamino carboxylate ligands, which forms an oblate spheroid with a 0.45 nm radius in the axial direction and 0.8 nm radius in the equatorial direction (Fig. 4 ▸). Indeed, the value obtained using a sphere form factor of 0.62 is intermediate between the two values and appears to be a good geometric approximation. A more accurate approximation of the overall cluster shape in solution would involve more sophisticated geometry and electron contrast models. However, in this experiment, the experimental SAXS signal does not allow such a precise description. With a better normalization that suffers less from the sample holder scattering signal, this limit may be overcome with an optimized setup in future work.

**Figure 5:**
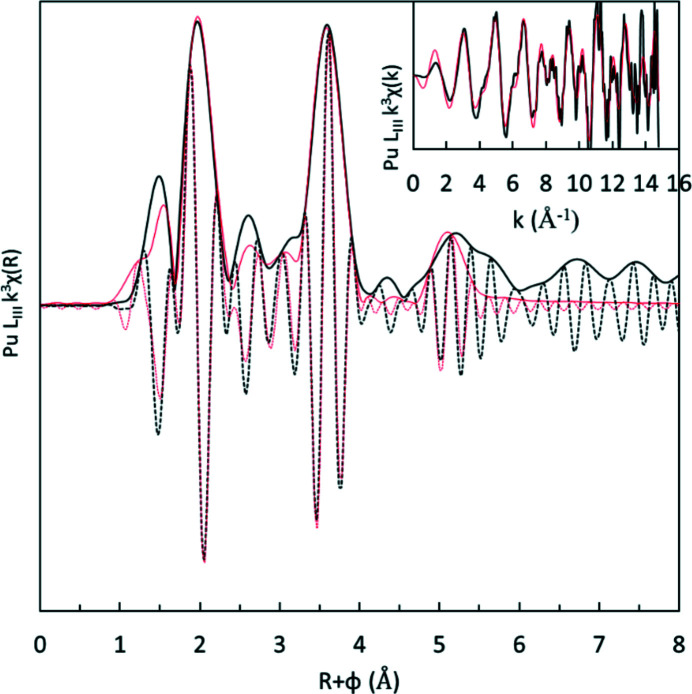
FT of the *k* ^3^-weighted EXAFS spectra (black lines) and best fit results (red lines) of the plutonium solutions prepared by dissolution of Pu^IV^ hexanuclear cluster crystals. The *k* ^3^-weighted EXAFS oscillations and fits are presented in the inset. The imaginary parts of the FT are the black and red dashed lines.

Previous XAS measurements carried out on the plutonium solution demonstrated the existence of the plutonium cluster as a soluble species. The clusters are made of six plutonium centres that form an octahedron, which are connected by oxo and hydroxo bonds alternating on each face of the octahedron and carboxylate functions that bond along the octahedron edges. The *k* ^3^-weighted EXAFS oscillations and the corresponding FT acquired from this experiment are presented in Fig. 5 ▸. The overall shape of the FT is consistent with previous measurements made on similar samples. Low-frequency contributions at *k* < 7 Å^−1^ correspond to the plutonium–oxygen coordination sphere (1 Å < *R*+ϕ < 2 Å in the FT). High-frequency oscillations at *k* > 7 Å^−1^ are dominated by a heavy backscatterer which shows a plutonium polynuclear species. An intermediate contribution observed between *R*+ϕ = 2.2 and 3.2 Å in the FT originates from carboxylate carbon atoms.

The best fit spectra (red lines in Fig. 5 ▸, and data in Table 3 ▸) were obtained using the crystal structure already published (Tamain *et al.*, 2016 ▸) and are in excellent agreement with the experimental data. In the fitting procedure, distances, DWFs, the amplitude reduction factor ( 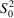 ) and the energy shift (*E*_0_) were refined while the coordination numbers were fixed according to the crystal structure. In agreement with the latter, the first actinide coordination shell is well reproduced by two distinct Pu–O subshells. The first one corresponds to two capping μ_3_-O^2−^ oxygen atoms at a distance of 2.18 Å (±−0.01 Å). The second Pu–O subshell at 2.40 Å contains the other six Pu—O distances (μ_3_-OH^−^, water molecules and O from carboxylate functions). As this subshell contains different types of bonds and distances, the DWF is large (0.008 Å^2^) compared with the short oxygen shell (0.004 Å^2^).

Considering the hexanuclear cluster geometry, the Pu—Pu signal splits into two shells. The edge-sharing shell consists of four Pu—Pu scattering paths with a 3.77 Å bond length. A longer bond corresponds to the opposite Pu atoms in the octahedron at 5.31 Å. Overall, all parameters are fully consistent with previous reports on this type of hexanuclear complex and agree well with the crystallographic structure.

## 4. Conclusions

The characterization of the Pu^IV^ hexanuclear cluster using synchrotron SAXS coupled with EXAFS undertakes an analytical challenge to describe the actinide coordination mode and structure in dilute solution. These species are of great interest for obvious environmental reasons and require a thorough characterization and speciation. Spectroscopic techniques and crystallographic characterization initially paved the way in this sense. In this experiment, we have extended our knowledge of these compounds by combining synchrotron X-ray scattering and absorption techniques. The synthesis of the hexanuclear cluster was controlled by UV–Vis–NIR spectroscopy and the EXAFS analyses were fully consistent with previous results. The SAXS results identify 1.2 nm-diameter spherical particles, in good agreement with the shape of the [Pu_6_(OH)_4_O_4_(H_2_O)_8_(HDOTA)_4_] complex described previously by single-crystal XRD.

The instrumental setup fulfils the expectations of users for measuring highly radioactive samples in environmentally relevant dilute conditions, even for weakly scattering clusters. Nevertheless, the instrument is being developed further. Thus, the MARS beamline is equipped with a Pilatus 3 2M (Dectris, Switzerland) hybrid photon-counting pixel detector having practically no detector readout noise, a high dynamic range and a short readout time. It is mounted on a new (*T_x_*, *T_s_*, *R_x_*) motorized support to form camera lengths anywhere from 0.35 to 3 m in 0.25 m steps. On the SAXS camera nose cone, a second detector affording partial 2D access to wide-angle X-ray scattering (WAXS) information will be operated to complement full 2D SAXS in the appropriate configuration. With careful beamline setup, these detectors allow overlap of the reduced 1D SAXS and WAXS data over the full camera length capability of the beamline. Thus, any reasonably sized sample environment can be accommodated whilst minimizing air gaps and attendant background scatter. To capitalize on the energy tunability of the beamline, anomalous SAXS might be exploited to probe changes in the scattering properties of selected atoms to gain information on the distribution of the resonant atoms within the sample.

## Figures and Tables

**Table 1:** X-ray scattering length density (ρ_c_) and electron contrast (Δρ^2^) used for SAXS diagram simulation calculations Values for the plutonium cluster take into account both the organic volume of the DOTA polyamino carboxylate ligands and the [Pu_6_(OH)_4_O_4_]^12+^ core.

	ρ_c_ (10^10^ cm^−2^)		Δρ^2^ (10^20^ cm^−4^)
Pu cluster	13.5	Pu cluster/H_2_O	16.6
H_2_O	9.43		

**Table 2:** Simulated results of SAXS diagrams obtained with *SasView* software *I*_0_ is the intensity for *Q* → 0, *R*_g_ is the radius of gyration and *R* is the radius of the sphere.

		Model		
Sample information		Guinier		Sphere
Name	*I*_0_ (cm^−1^)	*R*_g_ (nm)	*R* (nm)	*R* (nm)
Pu cluster	0.006 ± 0.003	0.48 ± 0.05	0.62 ± 0.05	0.62 ± 0.06

**Table 3:** EXAFS refined metrical parameters obtained for the best fit of the soluble Pu–DOTA hexanuclear clusters CN is the coordination number. Errors in brackets only reflect the mathematical uncertainty provided by the proposed model. Asterisks (*) indicate fixed values.

*E*_0_ = −0.9 (20) eV, *S*_0_^2^ = 0.75 (15)	Pu–μ_3_O^2−^	Pu–(μ_3_OH^−^, O_c_, H_2_O)	Pu–C	Pu–Pu1	Pu–Pu2
CN*	2*	6*	2*	4*	1*
σ^2^ (Å^2^)	0.004 (2)	0.008 (2)	0.003 (4)	0.006 (2)	0.003 (3)
*R* (Å)	2.18 (2)	2.40 (2)	3.35 (3)	3.77 (2)	5.3 (3)
*R*_XRD_ (Å)	2.20	2.36	3.34	3.76	5.31
